# Exploring the role of Epstein-Barr virus infection on the clinical features and survival in locally advanced cervical cancer: a retrospective cohort study

**DOI:** 10.3389/fonc.2024.1522244

**Published:** 2024-12-24

**Authors:** Denisse Castro-Uriol, Juana Vera, Bryan Valcarcel, Marco López-Ilasaca, Alejandro Yabar, Anaís Cámara, Luis Malpica, Brady Beltrán

**Affiliations:** ^1^ Departamento de Oncología y Radioterapia, Hospital Nacional Edgardo Rebagliati Martins, EsSalud, Lima, Peru; ^2^ Centro de Investigación de Medicina de Precisión, Universidad de San Martin de Porres, Lima, Peru; ^3^ Departmento de Anatomía Patológica, Hospital Nacional Edgardo Rebagliati Martins, EsSalud, Lima, Peru; ^4^ Department of Epidemiology, Milken Institute School of Public Health, The George Washington University, Washington, DC, United States; ^5^ Department of Medicine, Brigham and Women’s Hospital, Harvard Medical School, Boston, MA, United States; ^6^ Department of Lymphoma and Myeloma, The University of Texas MD Anderson Cancer Center, Houston, TX, United States

**Keywords:** Epstein-Barr virus, cervical cancer, clinical features, prognosis, survival

## Abstract

**Introduction:**

Epstein-Barr virus (EBV) infection has been linked to cervical cancer (CC), but few have described the clinical and outcome features of patients with CC and EBV infection.

**Methods:**

We conducted a single-center matched cohort study on 94 patients with CC. Real-time Polymerase chain reaction (RT-PCR) was used to detect *EBNA-1* (Epstein-Barr nuclear antigen 1) and *LMP-1* (Latent membrane protein 1). We used Kaplan-Meier and Cox regression analysis to evaluate the effect of EBV infection on overall survival (OS) and progression-free survival (PFS). Females with a positive EBV status were matched to those without infection using a propensity score.

**Results:**

Of the 94 patients in our cohort, 21 (22%) had a positive EBV status. Before and after matching, there were no differences in baseline clinical and sociodemographic features between patients diagnosed with CC with and without EBV infection. Most patients received concurrent chemoradiotherapy (73%) as frontline treatment. With a median follow-up of 67 months, the 5-year OS was 42% (95% CI: 33–55%) and the 5-year PFS was 37% (95% CI: 37–49%) in the entire population. Patients with EBV-positive status had comparable 5-year OS (50% vs. 37%, p-value=0.490; Hazard Ratio [HR] 0.77, 95% CI 0.36-1.62) and 5-year PFS (44% vs. 37%, p-value=0.750; HR 0.89, 95% CI 0.43-1.83) to those with EBV-negative CC, respectively.

**Conclusion:**

Females with CC and EBV infection have similar clinical features and outcomes compared to those without EBV infection.

## 1 Introduction

Cervical cancer (CC) is the fourth most common cause of cancer-related deaths among women and the fourth leading cause of death worldwide (1). Despite global advances, survival outcomes in Peru remain notably worse than those in Western countries (2–4). A previous Peruvian study found that locally advanced CC was the most common stage at presentation and was associated with poor outcomes, indicating that additional factors may be contributing to the unfavorable prognosis (4). This survival difference is generally associated with limited access to cancer care, resulting in a higher proportion of patients presenting with advanced-stage disease at diagnosis (4, 5). However, it is unclear whether other factors, such as environmental influences, including Epstein-Barr virus (EBV) infection, also contribute to these poor outcomes.

EBV-related cancers are more prevalent in Latin America than in other regions, with Peru showing one of the highest rates (6). EBV infection has been recognized as a prognostic factor in several cancers, though its impact on survival remains inconsistent across studies (7). In solid tumors, EBV-associated gastric carcinoma and nasopharyngeal carcinoma have generally been linked to favorable outcomes (8, 9). In contrast, EBV positivity in hematologic malignancies, such as diffuse large B-cell lymphoma treated with chemotherapy alone (10), natural killer/T-cell lymphomas (11), and EBV-positive recipients after allogeneic transplantation (12), have been associated with poorer survival. Our group recently reported the largest cohort of EBV-positive Diffuse large B-cell lymphoma in Latin America, demonstrating unfavorable clinical features but no significant survival differences when rituximab was incorporated into chemotherapy regimens (13). The influence of EBV on CC outcomes, however, remains largely unexplored.

Preclinical studies suggest that EBV may enhance and accelerate the integration of Human papillomavirus (HPV), thereby promoting tumor progression in cervical cancer and highlighting a possible synergistic interaction between these two oncogenic viruses (14). Yet, whether EBV infection leads to worse outcomes in locally advanced CC patients remains uncertain. A single-center study from China found no significant survival differences between EBV-positive and EBV-negative CC patients (15). However, this study focused predominantly on EBV prevalence, infection patterns, and certain pathological and immunological features without a detailed examination of key patient characteristics, treatment modalities, or comparative survival outcomes. Additionally, the study included a broader population, of which locally advanced cases were a minority. Herein, we aim to fill this gap by comprehensively characterizing the sociodemographic and clinical features, treatment approaches, and survival outcomes of CC patients with and without EBV infection in an endemic setting.

## 2 Materials and methods

### 2.1 Study design and population

We conducted a retrospective cohort study of adult females diagnosed with CC between December 2013 and June 2014, with follow-up through July 2019. The patients were identified using the Department of Oncology and Radiotherapy database of the Hospital Edgardo Rebagliati Martins in Lima, Peru. Medical records were manually reviewed, and data abstracted from August to September 2019 in a secured database. Inclusion criteria: females aged ≥18 years; cancer stage IIB, IIIA, or IIIB; and anatomopathological diagnosis of squamous cell carcinoma. We excluded those treated at outside healthcare centers, patients without medical records (lost or destroyed), patients with incomplete or insufficient data for pathological characterization, insufficient cervical tissue samples for real-time polymerase chain reaction (RT-PCR), and untreated patients or those who received non-platinum-based chemotherapy (n=95) with no intention to cure. The Institutional Review Board and Ethics Committee of the Hospital Edgardo Rebagliati Martins-EsSalud approved the conduction of this study.

### 2.2 Study variables

Baseline demographic and clinical features were abstracted at CC diagnosis. EBV positivity was defined as a positive result for the expression of Epstein-Barr nuclear antigen 1 (*EBNA-1*) (16) or latent membrane protein 1 (*LMP-1*) (17). Conversely, EBV negativity was defined as negative results for expressing *EBNA-1* and *LMP-1*. We collected data regarding the following sociodemographic and clinical covariates: age; parity; performance status; complete blood cell count (i.e., absolute leukocyte count, neutrophils, lymphocytes, monocytes, and platelets); red blood cell distribution width coefficient of variations and red blood cell distribution width standard deviation; serum albumin; clinical stage of CC; and frontline treatment received such as chemoradiotherapy (CRT) or radiotherapy (RT) only. Parity was classified as nulliparous (never given birth), primiparous (given birth once), and multiparous (given birth two or more times). Performance status was measured using the Eastern Cooperative Oncology Group (ECOG) scale. For CC staging, we used the *Fédération Internationale de Gynécologie et d’Obstétrique* staging system (18).

### 2.3 EBV analysis

Tissue specimens were obtained through an incisional biopsy of the cervical tumor site. They were routinely fixed in formalin and embedded in paraffin. The samples were stored in the hospital archives until subsequent analysis. Genomic DNA was purified from paraffin-embedded tissue sections. The following primers were used for RT-PCR: *EBNA-1* Forward 5'-TACAGGACCTGGAAATGGCC-3' and Reverse 5'-TCTTTGAGGT CCACT GC CG-3'; *LMP-1* Forward 5'-CAGTCAGGCAAGCCTATGA-3' and Reverse 5'-CTGGTT CCGGTGG AGATGA-3'. Additionally, the human β-actin gene (Forward: 5'- ATCATGTTTGAGACCTTCAACAC-3' and Reverse: 5'- CATCTCTTGCTCGAAGTCCAG-3') was used as an internal control for the presence of intact genomic DNA. A sample was considered EBV-positive when the fluorescence amplification curves were recorded by the thermal cycler as positive against *EBNA-1* and *LMP-1*.

### 2.4 Data analysis

Continuous variables were compared with a two-sample *t*-test or the Wilcoxon rank-sum test, as appropriate. We used the χ-squared test or Fisher’s exact test as appropriate for categorical variables. The median follow-up time was computed using the reverse Kaplan–Meier method. Our endpoints were overall survival (OS) and progression-free survival (PFS). OS was defined as the time (in months) from CC diagnosis until death from any cause, while PFS was determined from diagnosis until recurrence, death from any reason, or loss to follow-up, whichever comes first. Survival probabilities were estimated using the Kaplan-Meier method, and the curves were compared using the log-rank test.

The effect of EBV on OS and PFS was initially evaluated in multivariable Cox regression models, adjusting for age, parity, performance status, and cancer stage. To further reduce confounding, we matched females with EBV infection with those without infection using propensity score matching and a ratio of 2:1. We used the nearest neighborhood approach for the propensity score. The matched variables had an optimal standard mean difference: age (SMD=0.05), year of diagnosis (SMD=0.07), performance status (SMD=0.1), cancer stage (SMD=0.1), and frontline treatment (SMD=0.1). Subgroup analyses compared the effect of EBV on survival outcomes within the frontline treatment received (CRT or RT alone). Analyses are reported with hazard ratios (HRs) and 95% confidence intervals (CI). Analyses were performed in R using the packages “tidyverse,” “survival,” “survminer,” “MatchIt, “ and “ggplot2”.

## 3 Results

### 3.1 Patient characteristics

A total of 189 patients were assessed for eligibility, of whom 94 (49.7%) met the inclusion criteria. Patient characteristics at diagnosis are summarized in **Table 1**. The median age was 55 years. Most patients had ECOG 0–1 (70%), were multiparous (74%), and had clinical stage IIB (64%). A total of 21 (22.3%) patients had an EBV-positive status at CC diagnosis. According to frontline treatment, the majority received concurrent CRT (73%) compared to RT alone (27%).

**Table 1:** Clinical and treatment characteristics of patients with CC by EBV status before and after matching.

Characteristics No. (%) Median (range)	Entire cohort	Before matching			After matching		
		EBV Positive	EBV Negative	P-value	EBV Positive	EBV Negative	P-value
No. of patients	94	21	73		21	42	
Age at diagnosis	55 (28 - 86)	51 (30 - 83)	55 (28 - 86)	0.740	51 (30 - 83)	52 (29 - 86)	0.905
Age >50 years	55 (59)	11 (52)	44 (60)	0.518	11 (52)	22 (52)	1.000
Parity				0.300			0.286
0	5 (5)	0 (0)	5 (7)		0 (0)	3 (7)	
1	19 (20)	6 (29)	13 (18)		6 (29)	7 (17)	
2+	70 (74)	15 (71)	55 (75)		15 (71)	32 (76)	
ECOG score 2-4	28 (30)	9 (43)	19 (26)	0.137	9 (43)	9 (21)	0.076
FIGO stage				0.469			1.000
IIB	60 (64)	12 (57)	48 (66)		12 (57)	24 (57)	
IIIA/B	34 (36)	9 (43)	25 (34)		9 (43)	18 (43)	
Leucocytes, cells/µL	7860 (2500 - 21390)	6640 (2500 - 18780)	7960 (2670 - 21390)	0.494	6640 (2500 - 18780)	8025 (2670 - 21390)	0.468
Neutrophils, cells/µL	4690 (1360 - 16760)	4060 (1360 - 16220)	4960 (1770 - 16760)	0.678	4060 (1360 - 16220)	5385 (1770 - 16760)	0.610
Lymphocytes, cells/µL	1490 (230 - 5130)	1540 (230 - 3080)	1410 (260 - 5130	0.541	1540 (230 - 3080)	1405 (260 - 4110)	0.700
Monocytes, cells/µL	475 (110 - 1750)	490 (110 - 1130)	470 (120 - 1750)	0.635	490 (110 - 1130)	490 (120 - 1750)	0.627
Platelets, 10^3^ cells/µL	306 (149 - 857)	286 (183 - 646)	311 (149 - 857)	0.864	286 (183 - 646)	308 (163 - 745)	0.862
RDW CV, %	14 (12 - 23)	15 (12 - 22)	14 (12 - 23)	0.454	15 (12 - 22)	15 (12 - 23)	0.908
Frontline treatment approach				0.176			0.237
RT only	25 (27)	8 (38)	17 (23)		8 (38)	10 (24)	
CRT	69 (73)	13 (62)	56 (77)		13 (62)	32 (76)	
Chemotherapy cycles	5 (1 - 7)	5 (2 - 6)	5 (1 - 7)	0.969	5 (2 - 6)	6 (1 - 7)	0.971
RT sessions	25 (3 - 28)	25 (6 - 28)	25 (3 - 28)	0.638	25 (6 - 28)	25 (3 - 28)	0.722
Time to recurrence, months	14 (1 - 49)	14 (2 - 49)	10 (1 - 43)	0.754	10 (1 - 43)	10 (3 - 41)	0.812
Recurrence, at 5y	31 (33)	8 (38)	23 (32)	0.571	8 (38)	14 (33)	0.709
Mortality, at 5y	49 (52)	11 (52)	38 (52)	0.979	11 (52)	22 (52)	1.000

EBV, Epstein-Barr virus; ECOG, Eastern Cooperative Oncology Group; FIGO, Fédération Internationale de Gynécologie et d’Obstétrique; RDW-CV, red blood cell distribution width- coefficient of variation; CRT, Chemoradiotherapy; RT, radiotherapy.

### 3.2 Clinicopathological differences by EBV status

Before and after matching, there were no differences in baseline clinical and sociodemographic features between patients diagnosed with CC with and without EBV infection. CC patients with EBV infection tended to be younger (p-value=0.740), multiparous (p-value=0.3), exhibited ECOG scores of 2-3 (p-value=0.137), and were more likely to be in more advanced clinical stage (stages IIIA/B, p-value=0.469) compared to those without EBV infection (**Table 1**), but these findings were not statistically significant.

### 3.3 Survival outcomes

**Figure 1:**
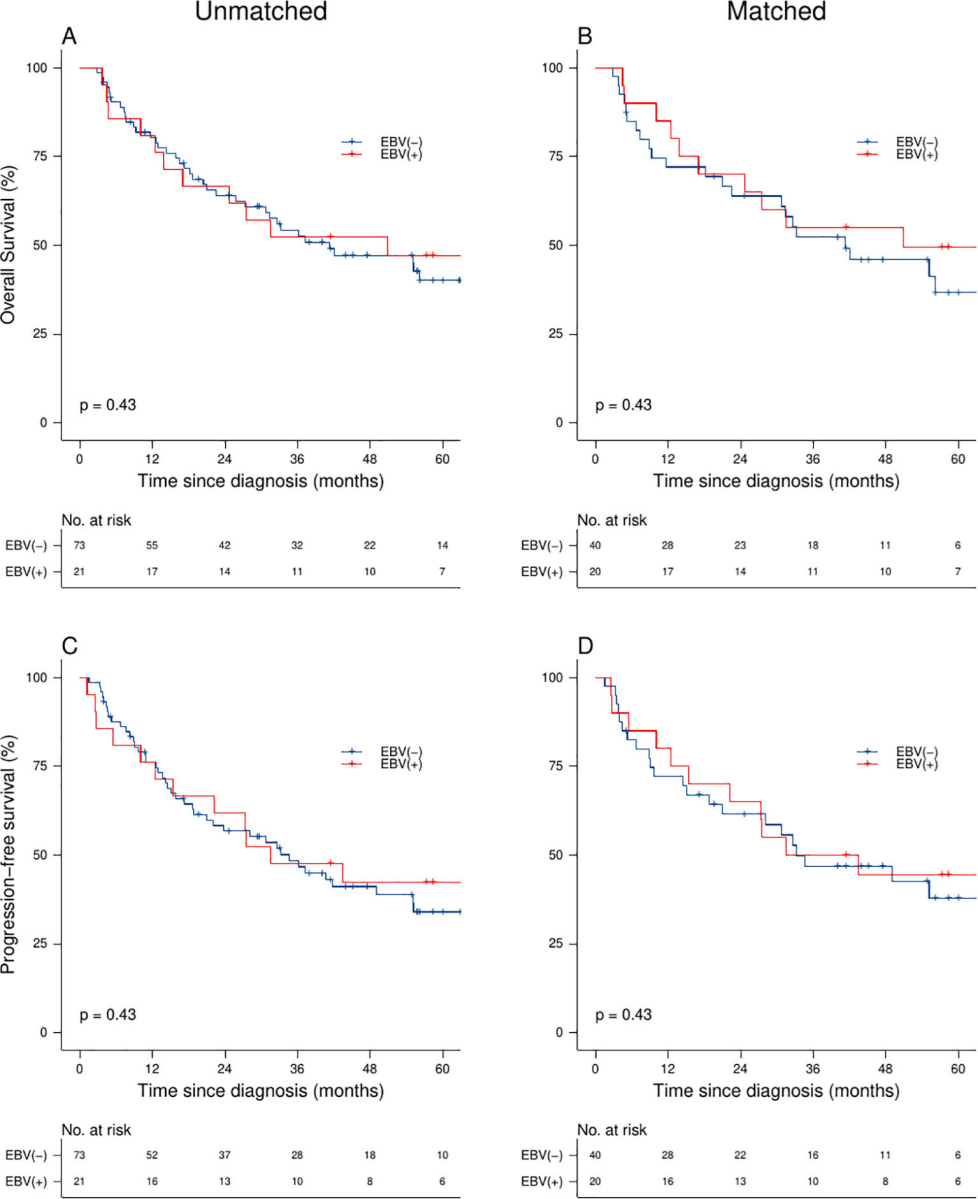
Survival analysis of CC patients: Overall survival **(A, B)** and progression-free survival **(C, D)** stratified by EBV status and matched cohort analysis.

The median follow-up was 67 months (95% CI: 56.0-66.9) in the entire cohort. The 5-year OS was 42% (95% CI: 33–55%) and the 5-year PFS was 37% (95% CI: 27–49%). In the unmatched cohort, EBV positivity showed similar outcomes in OS (5-year OS: 47% vs. 40%, p-value=0.780; adjusted HR [aHR] 0.85, 95% CI: 0.43-1.69), and PFS (5-year PFS: 42% vs. 34%, p-value=0.720; aHR 0.83, 95% CI: 0.43-1.60) compared to EBV negativity. Likewise, in the matched cohort, we identified that patients with CC and EBV infection had similar OS (5-year OS: 50% vs. 37%, p-value=0.490; aHR 0.77, 95% CI: 0.36-1.62) and PFS (5-year PFS: 44% vs. 37%, p-value=0.750; aHR 0.89, 95% CI: 0.43-1.83) compared to those without a positive EBV status (**Figure 1**, **Table 2**).

**Table 2:** Cox regression analyses of the effect of EBV status on the OS and PFS among patients with CC (**A**) before and (**B**) after matching.

	No. (%)	Overall survival		Progression-free survival	
		HR (95% CI)	aHR (95% CI)	HR (95% CI)	aHR (95% CI)
A. Unmatched					
EBV status					
Negative	73 (77.7)	1.00	1.00	1.00	1.00
Positive	21 (22.3)	0.91 (0.46-1.78)	0.85 (0.43-1.69)	0.89 (0.47-1.69)	0.83 (0.43-1.60)
Age group					
≤50	39 (41.5)	1.00	1.00	1.00	1.00
>50	55 (58.5)	1.44 (0.79-2.62)	1.23 (0.66-2.27)	1.61 (0.91-2.87)	1.37 (0.76-2.48)
Parity					
0-1	24 (25.5)	1.00	1.00	1.00	1.00
2+	70 (74.5)	1.84 (0.86-3.93)	1.57 (0.72-3.44)	1.83 (0.89-3.74)	1.49 (0.71-3.12)
FIGO stage					
IIB	60 (63.8)	1.00	1.00	1.00	1.00
IIIA/B	34 (36.2)	1.79 (1.02-3.16)	1.68 (0.94-3.02)	1.95 (1.14-3.34)	1.83 (1.05-3.17)
B. Matched					
EBV status					
Negative	42 (66.7)	1.00	1.00	1.00	1.00
Positive	21 (33.3)	0.92 (0.44-1.89)	0.89 (0.43-1.84)	0.90 (0.45-1.81)	0.87 (0.43-1.75)
Age group					
≤50	30 (47.6)	1.00	1.00	1.00	1.00
>50	33 (52.4)	1.61 (0.79-3.28)	1.25 (0.57-2.75)	1.96 (0.98-3.94)	1.49 (0.69-3.21)
Parity					
0-1	16 (25.4)	1.00	1.00	1.00	1.00
2+	47 (74.6)	1.80 (0.74-4.37)	1.49 (0.57-3.92)	2.12 (0.88-5.10)	1.61 (0.62-4.17)
FIGO stage					
IIB	36 (57.1)	1.00	1.00	1.00	1.00
IIIA/B	27 (42.9)	1.67 (0.84-3.31)	1.50 (0.74-3.05)	1.75 (0.91-3.37)	1.50 (0.76-2.94)

aHR, adjusted Hazard ratio; CI, confidence interval; EBV, Epstein-Barr virus; FIGO, Fédération Internationale de Gynécologie et d’Obstétrique; HR, hazard ratio.

**Figure 2:**
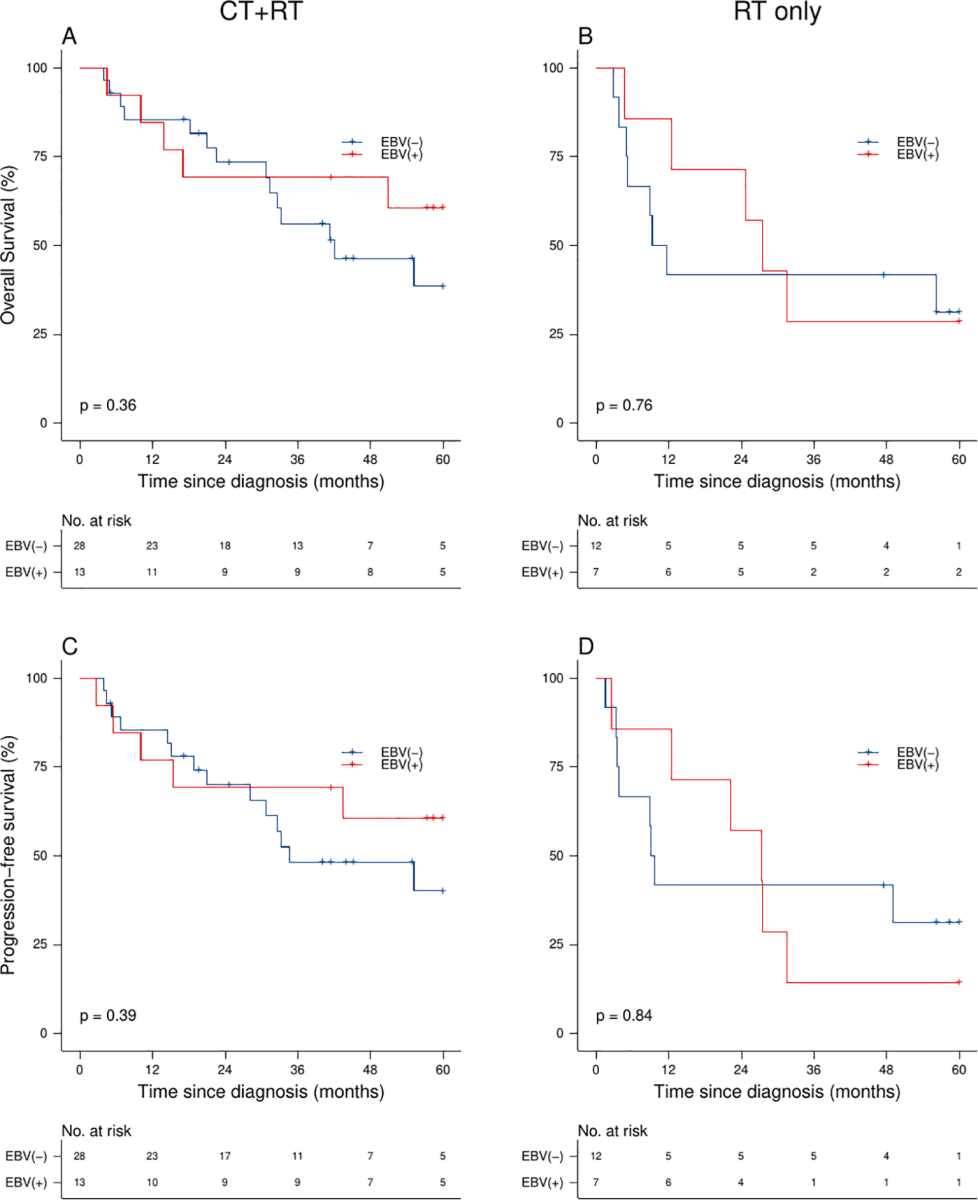
Survival analysis of CC patients: Overall survival **(A, B)** and progression-free survival **(C, D)** stratified by EBV status and frontline treatment modality.

In a subgroup analysis, the 5-year OS rates were 34% (95% CI: 19-60%) for RT and 45% (95% CI: 34-60%) for CRT, with a p-value of 0.121. The 5-year PFS rates were 26% (95% CI: 13-52%) for RT and 40% (95% CI: 29-56%) for CRT, with a p-value of 0.069. We did not identify differences in OS and PFS between patients with CC with and without EBV infection who received either CRT (5-year OS: 61% vs. 39%, p-value=0.360; and 5-year PFS: 61% vs. 40%, p-value=0.390) or RT (5-year OS: 29% vs. 31%, p-value=0.760; and 5-year PFS: 14% vs. 31%, p-value=0.840) as a frontline treatment (**Figure 2**).

## 4 Discussion

This study is among the first to comprehensively explore the impact of EBV infection on survival in women with CC living in an endemic Latin American country like Peru. We observed no significant differences in clinical features or survival outcomes between patients diagnosed with CC with and without EBV infection.

Although we were unable to find a prognostic role for EBV, existing evidence supports that EBV plays an important role in tumor progression in HPV-related CC (14). A meta-analysis of retrospective studies from Asia, Europe, Africa, and Latin America found differences in the rates of EBV infection among cervical lesions. The highest prevalence was observed in carcinoma cases (43.6%), compared to preinvasive disease (cervical intraepithelial neoplasia grade 1: 23%, and cervical intraepithelial neoplasia grade 2 and 3: 34%) and normal cervical samples (19%) (19). Notably, Latin America had the highest EBV prevalence among carcinoma cases (62%) (19). The most representative study from the meta-analysis was conducted in Brazil, where 169 CC cases were examined. They found that EBV was higher in CC (64.2%) compared to high-grade lesions (21.1%) and normal cervical samples (8.9%) (20). Similarly, a recent study of Chinese women with CC reported an EBV prevalence of 20.2% (15). These findings indicate that patients with EBV infection have a higher frequency of carcinoma than those without EBV infection and likely suggest that the prevalence of EBV may differ by region, with Latin America exhibiting one of the highest percentages.

Our study did not show significant differences in clinical features or frontline treatment approaches between patients with and without EBV infection. Interestingly, although our population differed from that of Zuo et al., who included a broad range of patients with both early and advanced clinical stages of CC, their findings showed that patients with EBV infection and CC had significantly more advanced clinical stages (≥ IIB) (52.4% vs. 10.8%, p-value <0.01), a higher rate of tumor‐positive lymph nodes (61.9% vs. 26.5%, p-value=0.02), neural invasion (38.1% vs. 14.5%, p-value =0.014), and increased infiltration depth (1.2 vs. 0.9 cm, p-value=0.031) compared to patients without EBV infection (15). Additionally, a significant increase in immunosuppressive cells such as FoxP3^+^, CTL4^+^, ratio of Tregs cells to CD8+ tumor-infiltrating lymphocytes, PDL-1, and PD-1 expression was observed in EBV-positive than EBV‐negative squamous CC cases (15), indicating that EBV-positive tissue may be more susceptible to immunotherapy (21).

To date, only one study evaluated the impact of EBV on survival outcomes in CC. Similar to our findings, Zuo et al. found no significant association between EBV positivity in OS (p-value=0.212) or PFS (p-value=0.667) in patients with CC (15). One possible explanation for both our findings and those of Zuo et al. is that EBV may serve only as an enhancer of HPV, facilitating the transition from normal tissue to invasive cancer rather than serving as a prognostic factor, as observed in other malignancies (14, 22). Our results may have been influenced by delays in treatment initiation at our institution, obscuring differences between groups, as not all patients received timely and appropriate frontline treatment. This effect likely explains the inferior survival outcomes in our cohort compared to previous reports, where survival rates ranged from 60-80% in developed countries (2, 15) and 50-60% in developing countries (2). These results may be attributed to barriers to access treatment that affect up to 66% of the population in Peru (5).

Our study has limitations. First, the retrospective design restricted the availability of complete clinical and pathological data, likely introducing bias and confounding factors and limiting the strength of the associations observed. Second, we excluded 95 patients due to tissue deterioration, which may have affected the generalizability of our results; however, we do not expect to be differential based on EBV status. Third, this study was conducted in a single center that primarily serves the insured working population through the Social Security System in Peru, limiting the extrapolation of our findings to this specific demographic. Additionally, HPV status was not directly assessed; however, given the nearly universal presence of HPV in squamous cell carcinoma histology (23), it is likely that all patients with squamous cell carcinoma were HPV-positive. Despite these limitations, the main strength of our study lies in the use of RT-PCR, a highly sensitive technique for detecting EBV in the tissues of seropositive individuals (24). While the gold standard for EBV detection (EBER study) is effective for identifying latent infections, it has limitations in detecting cases where the virus is in the lytic phase, resulting in lower sensitivity and a higher rate of false-negative results compared to RT-PCR (25).

In conclusion, patients with CC and EBV infection in our Peruvian cohort demonstrated clinical features and survival outcomes comparable to those without EBV infection. Consequently, the routine screening for EBV in CC remains uncertain and requires further investigation. Future multicenter prospective studies are essential to clarify the prognostic significance of EBV infection in CC and determine its potential role in guiding clinical management.

## Data Availability

The raw data supporting the conclusions of this article will be made available by the authors, without undue reservation.
